# Quantification of myocardial blood flow with cardiovascular magnetic resonance throughout the cardiac cycle

**DOI:** 10.1186/s12968-015-0107-3

**Published:** 2015-01-29

**Authors:** Manish Motwani, Ananth Kidambi, Akhlaque Uddin, Steven Sourbron, John P Greenwood, Sven Plein

**Affiliations:** Leeds Institute of Cardiovascular and Metabolic Medicine, Division of Biomedical Imaging, University of Leeds, Leeds, UK; Division of Medical Physics, University of Leeds, Leeds, UK

**Keywords:** Cardiovascular magnetic resonance imaging, Myocardial perfusion imaging, Myocardial blood flow

## Abstract

**Background:**

Myocardial blood flow (MBF) varies throughout the cardiac cycle in response to phasic changes in myocardial tension. The aim of this study was to determine if quantitative myocardial perfusion imaging with cardiovascular magnetic resonance (CMR) can accurately track physiological variations in MBF throughout the cardiac cycle.

**Methods:**

30 healthy volunteers underwent a single stress/rest perfusion CMR study with data acquisition at 5 different time points in the cardiac cycle (early-systole, mid-systole, end-systole, early-diastole and end-diastole). MBF was estimated on a per-subject basis by Fermi-constrained deconvolution. Interval variations in MBF between successive time points were expressed as percentage change. Maximal cyclic variation (MCV) was calculated as the percentage difference between maximum and minimum MBF values in a cardiac cycle.

**Results:**

At stress, there was significant variation in MBF across the cardiac cycle with successive reductions in MBF from end-diastole to early-, mid- and end-systole, and an increase from early- to end-diastole (end-diastole: 4.50 ± 0.91 vs. early-systole: 4.03 ± 0.76 vs. mid-systole: 3.68 ± 0.67 vs. end-systole 3.31 ± 0.70 vs. early-diastole: 4.11 ± 0.83 ml/g/min; all p values <0.0001). In all cases, the maximum and minimum stress MBF values occurred at end-diastole and end-systole respectively (mean MCV = 26 ± 5%). There was a strong negative correlation between MCV and peak heart rate at stress (r = −0.88, p < 0.001). The largest interval variation in stress MBF occurred between end-systole and early-diastole (24 ± 9% increase). At rest, there was no significant cyclic variation in MBF (end-diastole: 1.24 ± 0.19 vs. early-systole: 1.28 ± 0.17 vs.mid-systole: 1.28 ± 0.17 vs. end-systole: 1.27 ± 0.19 vs. early-diastole: 1.29 ± 0.19 ml/g/min; p = 0.71).

**Conclusion:**

Quantitative perfusion CMR can be used to non-invasively assess cyclic variations in MBF throughout the cardiac cycle. In this study, estimates of stress MBF followed the expected physiological trend, peaking at end-diastole and falling steadily through to end-systole. This technique may be useful in future pathophysiological studies of coronary blood flow and microvascular function.

## Background

Myocardial blood flow (MBF) varies throughout the cardiac cycle in response to changes in myocardial tension and phasic compression of the myocardial microcirculation [1-4]. The squeezing effect of myocardial contraction causes arterial blood inflow to peak during diastole when myocardial tension is low, and venous outflow to peak during systole when myocardial tension is high. Diseases such as diabetes, atherosclerosis, cardiomyopathies, and arterial hypertension result in functional and morphologic microvascular changes, which may precede clinical signs and symptoms [5]. Quantitative assessment of MBF and characterization of cyclic myocardial perfusion variation in these diseases may offer valuable additional information relating to microvascular integrity and function.

Over the last decade, several animal, normal volunteer and patient studies have validated the use of cardiovascular magnetic resonance (CMR) for absolute MBF quantification against microsphere and invasive coronary flow reserve measurements [6-8]. With conventional 2D perfusion CMR methods, data are acquired in a different cardiac phase for each slice. Previous volunteer and patient studies have now shown a significant difference in MBF quantified using CMR between acquisition in systole and diastole, suggesting that cardiac phase needs to be considered when interpreting CMR-derived MBF values [9-11]. Although the development of 3D perfusion CMR, which allows the specific phase of data acquisition to be chosen, may overcome this concern in the future, these differences remain a challenge for CMR perfusion. However, they also provide the opportunity to interrogate cyclic variations in perfusion as a unique diagnostic tool, not assessable by positron-emission tomography, which assesses myocardial perfusion cumulatively.

So far, temporal and spatial constraints on dynamic CMR perfusion imaging have restricted previous volunteer and patient studies to assessing MBF between only two different time points in the cardiac cycle, and it is therefore unknown whether CMR has the capability to track changes in MBF *throughout* the cardiac cycle [9-11]. Advanced acceleration techniques, based on spatiotemporal undersampling, which have mostly been employed to achieve higher spatial resolution, can also be used to improve temporal resolution in perfusion CMR studies. The purpose of the study was to capitalise on the dynamic aspect of CMR perfusion data acquisition and assess whether quantitative perfusion CMR can accurately follow the expected physiological variation in MBF *throughout* the cardiac cycle.

## Methods

### Study population

Thirty-three healthy volunteers were recruited. Exclusion criteria included any history of cardiovascular disease, diabetes mellitus, hypertension, hyperlipidemia, smoking or any contraindications to CMR, adenosine, or gadolinium-based contrast agents. The study protocol was approved by the local ethics committee, and written informed consent was obtained from all volunteers. All volunteers were instructed to refrain from caffeine for 24 hours before their CMR study.

### CMR protocol

**Figure 1:**
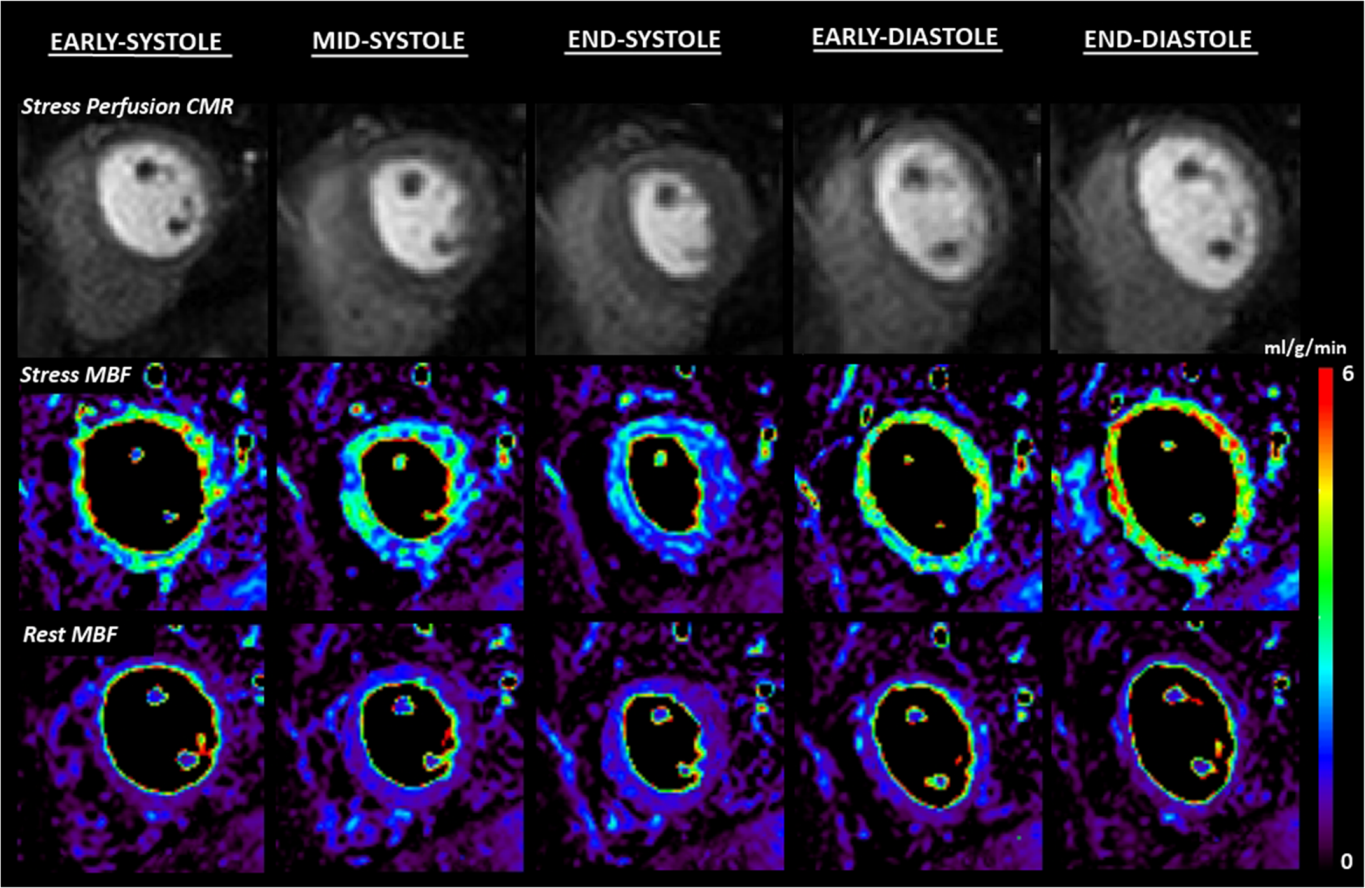
**Case example: quantitative perfusion CMR throughout the cardiac cycle.** This example shows stress perfusion CMR (top row) in a healthy volunteer acquired at 5 different time points throughout the cardiac cycle in a single mid-ventricular slice facilitated by 8-fold *k-t* BLAST acceleration. Corresponding stress and rest MBF estimates are shown as myocardial maps (middle and bottom row respectively). Stress MBF shows significant cyclic variation peaking in end-diastole and steadily falling throughout systole. The maximal interval change in stress MBF is seen between end-systole and early-diastole. No significant cyclic variation was seen in rest MBF. CMR = cardiovascular magnetic resonance; BLAST = broad linear acquisition speed-up technique; MBF = myocardial blood flow.

All volunteers underwent a single stress and rest perfusion CMR study performed on a 1.5 T scanner (Intera, Philips Healthcare, Best, the Netherlands) equipped with a five-element cardiac phased array receiver coil. Perfusion data were acquired in the same single mid-ventricular 2D slice at 5 different time points in each R-R interval (early-systole, mid-systole, end-systole, early-diastole and end-diastole) facilitated by *k-t* broad-use linear acquisition speed-up technique (*k-t* BLAST) acceleration (Figure 1). Details of the perfusion pulse sequence were as follows: 2D saturation recovery gradient-echo sequence accelerated with 8-fold *k-t* BLAST and 11 interleaved training profiles, no partial Fourier or partial echo acquisition, TR 3.4 ms, TE 1.7 ms, flip angle 15°, one saturation pre-pulse per slice (i.e. per time point), image acquisition time per slice 103 ms, matrix 192 × 192, median FOV 310 mm and in-plane spatial resolution 1.6 × 1.6 mm.

Vertical and horizontal long-axis cine images were used to identify appropriate trigger delays for each of the 5 time points [9-12]. Additionally, because of the longitudinal lengthening of the heart from systole to diastole, the position of the mid-ventricular perfusion slice at each time point was individually planned from the chosen end-diastolic, early-systolic, mid-systolic, end-systolic and early-diastolic cine frames [9-11].

Stress perfusion images were acquired during intravenous adenosine-induced hyperemia (140 mcg/kg/min administered for 4 min). An intravenous bolus of 0.05 mmol/kg gadopentetate dimeglumine (Magnevist, Schering, Germany) was administered at a rate of 4.0 ml/s followed by a 20 ml saline flush. Stress perfusion CMR was followed by cine imaging covering the left ventricle in short-axis sections. Rest perfusion CMR was performed 15 min after stress, using identical imaging parameters. Late gadolinium enhancement (LGE) imaging was not performed.

### Image quality

Perfusion images were reviewed for image quality by 2 observers acting in consensus (M.M. and A.K., 3 yrs and 2 yrs experience respectively). Image quality was scored as follows: 0 = non-diagnostic, 1 = poor, 2 = adequate and 3 = excellent. The occurrence of artifact related to respiratory-motion, *k-t* reconstruction or dark-rim artifact was scored as follows: 0 = none, 1 = mild, 2 = moderate and 3 = severe.

### MBF estimation

Perfusion images were processed offline using previously validated in-house software (PMI 0.4; written in IDL 6.4 (ITT Visual Information Systems, Boulder, CO) [11,13].

#### Per-subject analysis

Following manual rigid motion-correction, a circular region of interest (ROI) was drawn in the LV cavity at end-diastole, to derive the arterial input function (AIF). The same (end-diastolic) AIF was used for all estimates of MBF in order to avoid potential variations in the AIF between phases with subsequent effects on MBF estimation [9-11].

A whole-slice myocardial ROI excluding any dark-rim artifact was drawn on perfusion images for each of the 5 time points. Signal intensity–time data were converted to concentration-time data by subtracting the baseline signal. Global MBF was estimated at stress and rest using constrained deconvolution with a delayed Fermi-model applied to the first pass [14]. Myocardial perfusion reserve (MPR) was calculated as stress MBF divided by rest MBF.

Interval variations in MBF or MPR between successive time points were expressed as percentage change. Maximal cyclical variation (MCV) in MBF was calculated as the percentage difference between maximum and minimum values in a cardiac cycle.

#### Per-territory analysis

The above analysis was repeated on a per territory basis by segmenting the perfusion slices according to the 17-segment American Heart Association model [15]. For each perfusion territory, a myocardial ROI including all segments pertaining to that territory was outlined. MBF and MPR estimates at each time point were obtained using the same algorithms as for the per subject analysis.

#### Reproducibility

Per-subject analysis was repeated on perfusion data from ten random volunteers 1 month later by the same observer (M.M.) and by a second observer A.K. (3 yrs and 2 yrs experience respectively). A.K. was blinded to the results of all previous analyses.

### Statistical analysis

Analysis was performed using SPSS 17.0 (SPSS, Chicago, IL). Data are presented as mean ± SD. Mean perfusion values (MBF and MPR) were compared at the 5 different time points using one-way repeated measures analysis of variance (ANOVA) with Greenhouse-Geisser correction for multi-sample sphericity and Bonferonni adjustment for post-hoc analysis. Mean perfusion values at each time point were compared between perfusion territories using standard ANOVA. Intra- and inter-observer reproducibility for MBF, MPR and MCV were assessed by calculating coefficients of variation (CoVs): SD of the differences divided by the mean. All statistical tests were two-tailed and a p value <0.05 was considered significant.

## Results

### Study population

Thirty-three healthy volunteers were recruited. One volunteer could not complete the scan due to claustrophobia. Data from 2 other volunteers were excluded due to technical problems preventing analysis (1 excessive heart rate variability, 1 mistimed contrast injection). Data from a total of 30 volunteers (90 perfusion territories) were therefore available for the final analysis. Clinical details of the 30 study volunteers (18 men; mean age 22 ± 2 yrs) are summarized in Table 1.

**Table 1:** **Healthy volunteer demographics**

**Parameter**	**Data (n = 30)**
**Age (yrs)**	22 ± 2
**Sex, n (%)**	
Male	18 (60)
Female	12 (40)
**LV function**	
EF %	61 ± 5
EDV, ml	140 ± 26
ESV, ml	59 ± 14
LV Mass, g/m^2^	82 ± 22
**Hemodynamics at Peak Stress**	
Heart rate (beats/min)	81 ± 9
Systolic blood pressure (mmHg)	127 ± 20
RPP (mmHg x beats/min)	10226 ± 2319

Data n ± SD. LV = left ventricle; EDV = end-diastolic volume; ESV = end-systolic volume; RPP = rate-pressure product.

### Image quality

Perfusion images for all 30 volunteers were of analyzable quality. Image quality was graded as excellent overall (median score = 3) and there was negligible artefact (median score = 0). In 2 subjects (7%), perfusion images were affected by *k-t* reconstruction artifacts at stress and/or rest due to respiratory motion, but in both cases, these artifacts occurred at the end of the breath-hold and did not affect analysis of the first-pass perfusion images.

### MBF estimation

#### Per subject analysis

**Figure 2:**
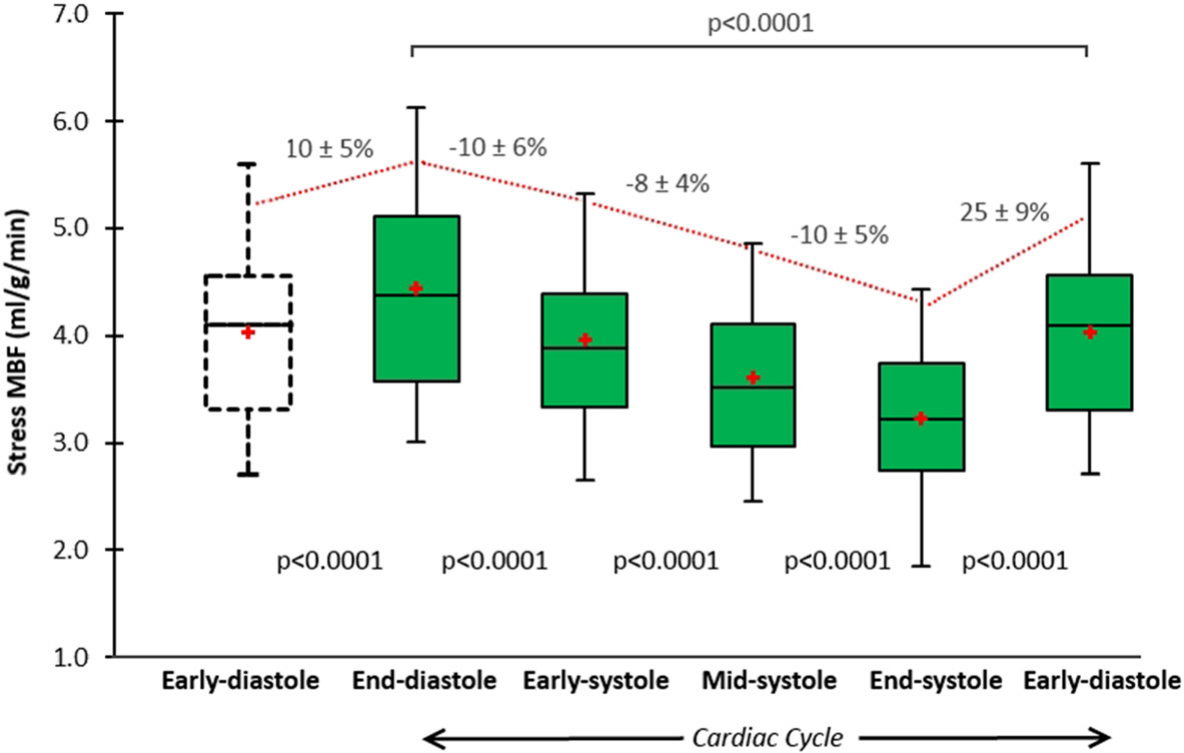
**Cyclic variation in stress MBF.** Mean stress MBF in healthy volunteers (n = 30) showed significant cyclic variation throughout the cardiac cycle (p < 0.0001). Box plots for stress MBF show the interquartile range (box), median (dividing black line) and mean (red cross) with whiskers extending to 1.5 x interquartile range. There were successive reductions in stress MBF from end-diastole to early-, mid- and end-systole, and a significant increase from early- to end-diastole (all p values <0.0001) (trend shown by red line). The maximal interval change in stress MBF was between end-systole and early-diastole (25% increase). MBF = myocardial blood flow.

**Figure 3:**
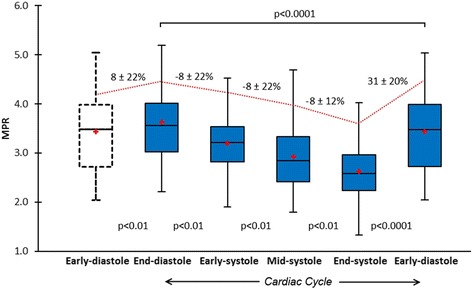
**Cyclic variation in MPR.** Mean MPR in healthy volunteers (n = 30) showed significant cyclic variation throughout the cardiac cycle (p < 0.0001). Box plots for MPR show the interquartile range (box), median (dividing black line) and mean (red cross) with whiskers extending to 1.5 x interquartile range. There were successive reductions from end-diastole to early-, mid- and end-systole, and a significant increase from early- to end-diastole (all p values <0.01) (trend shown by red line). The maximal interval change in MPR was between end-systole and early diastole (31% increase). MPR = myocardial perfusion reserve.

**Figure 4:**
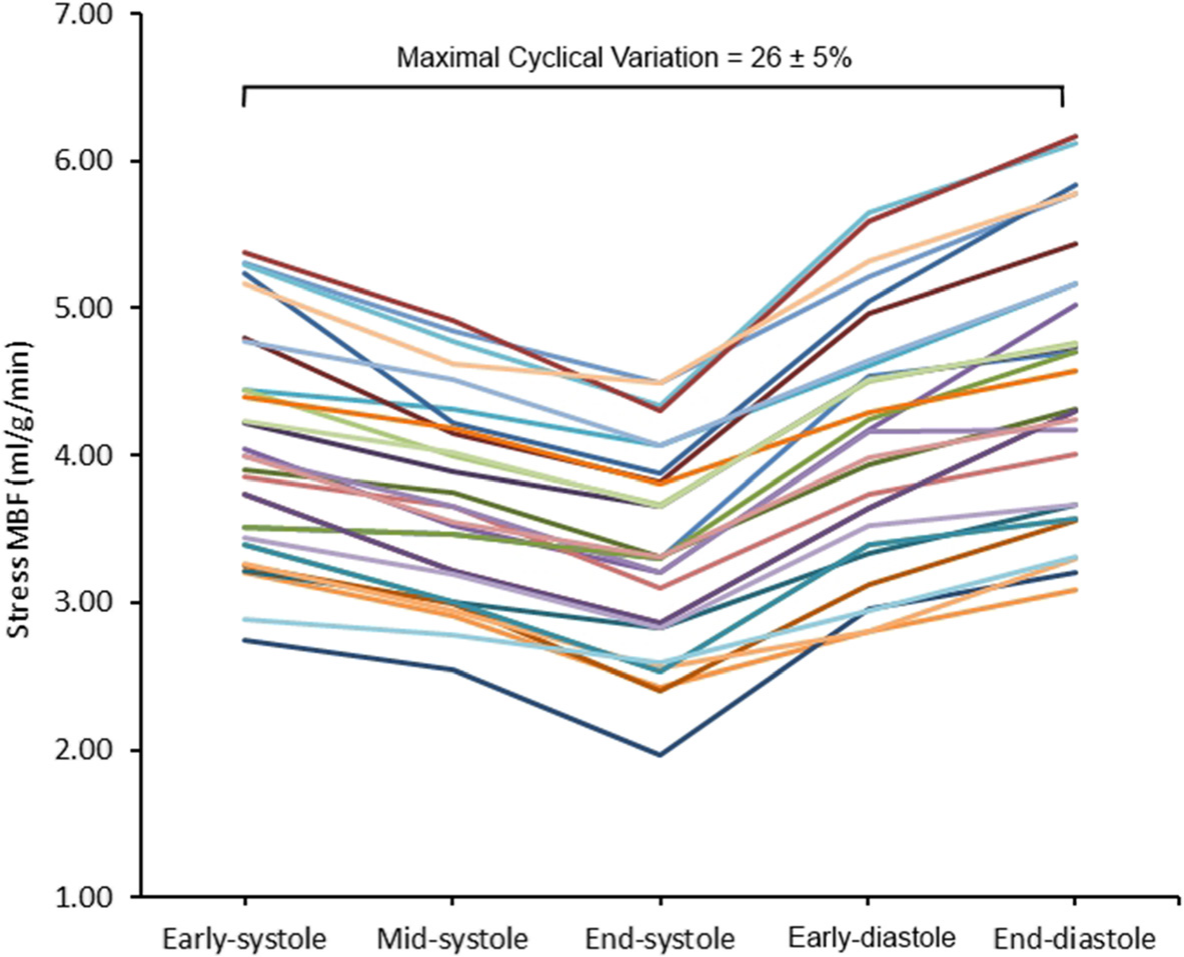
**Individual stress MBF estimates in healthy volunteers.** There was significant cyclic variation in stress MBF in all volunteers (n = 30). In all cases the peak MBF occurred at end-diastole and the minimum at end-systole. The mean maximal cyclic variation in stress MBF was 26%. MBF = myocardial blood flow.

**Figure 5:**
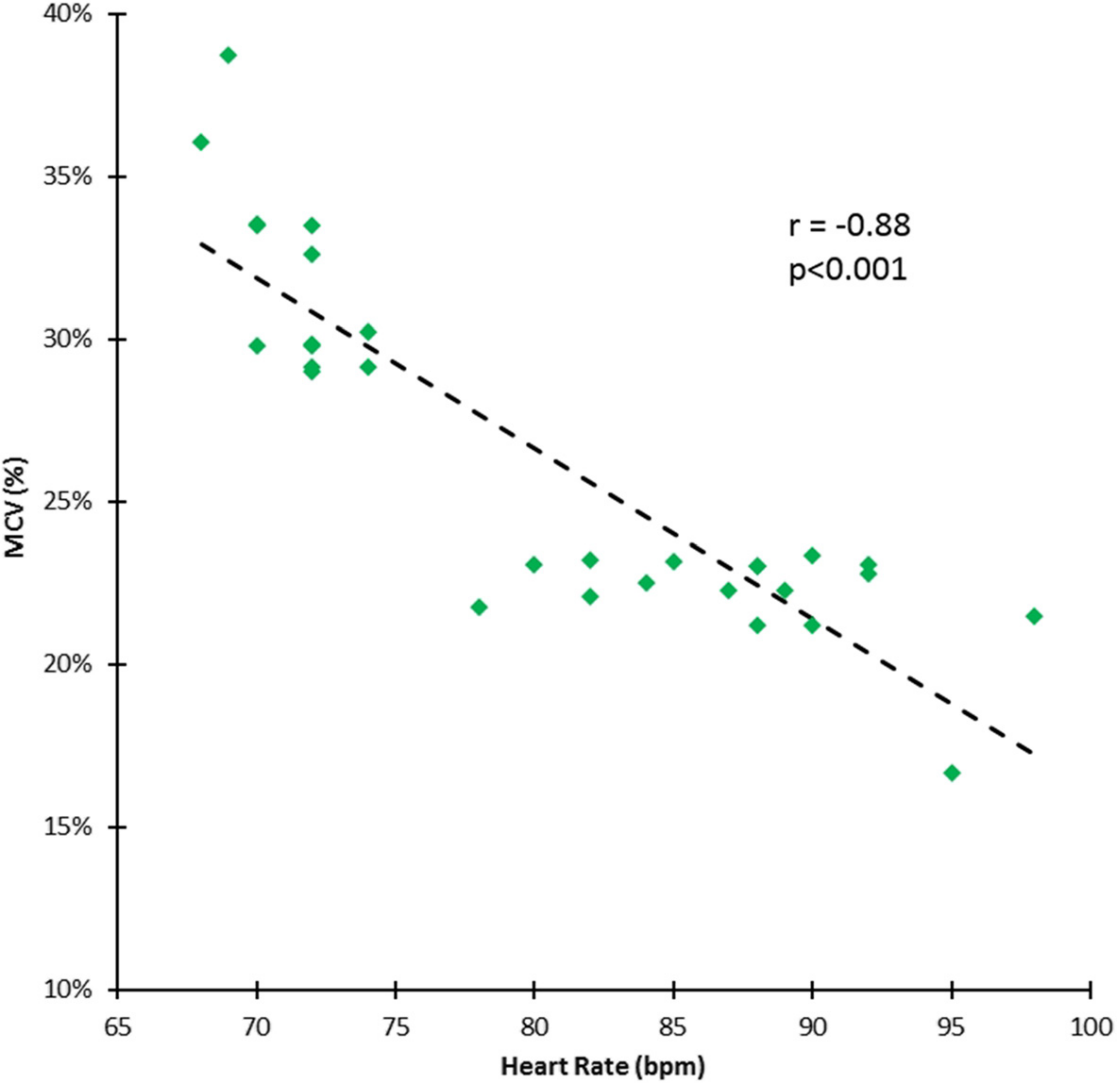
**Correlation between MCV and peak heart rate at stress.** There was a strong linear negative correlation between the maximal cyclic variation (MCV) in stress MBF and the peak heart rate during adenosine-induced maximal hyperemia in healthy volunteers (n = 30). MBF = myocardial blood flow.

Estimates of MBF and MPR at each of the 5 time points in the cardiac cycle are seen in Table 2. There was significant cyclic variation in stress MBF (p < 0.0001) and MPR (p < 0.0001) with successive reductions from end-diastole to early-, mid- and end-systole, followed by an increase from early- to end-diastole (all post-hoc p values <0.01) (Table 2, Figures 1, 2, 3 and 4). In all cases, the maximum and minimum stress MBF values occurred at end-diastole and end-systole respectively with a mean MCV of 26 ± 5% (Figure 4). There was a strong negative correlation between MCV and peak heart rate at stress (r = −0.88, p < 0.001) (Figure 5). The largest interval variation in stress MBF occurred between end-systole and early-diastole (24 ± 9% increase) (Figure 2). The largest interval variation in MPR occurred between end-systole and early-diastole (31 ± 20% increase) (Figure 3). At rest, there were no significant cyclical variations in MBF (p = 0.71) (Table 2, Figure 1).

**Table 2:** **Estimates of MBF and MPR throughout the cardiac cycle**

	***End-diastole***	***Early-systole***	***Mid-systole***	***End-systole***	***Early-diastole***	***P***
**Global**						
* Stress MBF*	4.50 ± 0.91	4.03 ± 0.76	3.68 ± 0.67	3.31 ± 0.69	4.11 ± 0.83	p < 0.0001
* Rest MBF*	1.24 ± 0.19	1.28 ± 0.17	1.28 ± 0.17	1.27 ± 0.19	1.29 ± 0.19	p = 0.71
* MPR*	3.63 ± 0.95	3.19 ± 0.66	2.92 ± 0.66	2.62 ± 0.63	3.40 ± 0.92	p < 0.0001
LAD perfusion territory*						
* Stress MBF*	4.38 ± 0.87	3.88 ± 0.78	3.59 ± 0.64	3.19 ± 0.67	3.99 ± 0.80	p < 0.0001
* Rest MBF*	1.25 ± 0.16	1.30 ± 0.14	1.24 ± 0.11	1.25 ± 0.13	1.21 ± 0.13	p = 0.14
* MPR*	3.54 ± 0.74	3.01 ± 0.60	2.91 ± 0.53	2.58 ± 0.58	3.35 ± 0.80	p < 0.0001
LCX perfusion territory*						
* Stress MBF*	4.40 ± 0.94	3.93 ± 0.70	3.58 ± 0.67	3.21 ± 0.69	4.01 ± 0.85	p < 0.0001
* Rest MBF*	1.25 ± 0.08	1.28 ± 0.17	1.24 ± 0.14	1.24 ± 0.14	1.23 ± 0.12	p = 0.60
* MPR*	3.52 ± 0.73	3.12 ± 0.65	2.93 ± 0.63	2.60 ± 0.56	3.29 ± 0.85	p < 0.0001
RCA perfusion territory*						
* Stress MBF*	4.55 ± 0.90	4.13 ± 0.76	3.78 ± 0.67	3.41 ± 0.69	4.21 ± 0.85	p < 0.0001
* Rest MBF*	1.33 ± 0.15	1.38 ± 0.17	1.38 ± 0.20	1.39 ± 0.19	1.33 ± 0.19	p = 0.50
* MPR*	3.46 ± 0.78	3.03 ± 0.60	2.78 ± 0.60	2.49 ± 0.57	3.24 ± 0.83	p < 0.0001

*Additionally there were no significant differences in mean perfusion values (MBF or MPR) between territories at any point in the cardiac cycle (all p values >0.05). MBF = myocardial blood flow in ml/min/g; MPR = myocardial perfusion reserve; LAD = left anterior descending; LCX = left circumflex; RCA = right coronary artery.

#### Per territory analysis

Estimates of MBF and MPR in each perfusion territory at each of the 5 time points in the cardiac cycle are seen in Table 2. There were no significant differences in stress MBF, rest MBF or MPR between perfusion territories at each of the 5 cardiac phases assessed (all p values > 0.05) (Table 2). There was significant cyclic variation in stress MBF and MPR in all 3 perfusion territories (all p values <0.0001) (Table 2). MCV for stress MBF was similar in all perfusion territories (left anterior descending [LAD]: 27 ± 8% vs. left circumflex [LCX]: 27 ± 5% vs. right coronary artery [RCA]: 25 ± 5%; p = 0.45). The largest interval variation in the LAD, LCX and RCA perfusion territories was the increase in stress MBF and MPR between end-systole and early-diastole and the magnitude was similar in all 3 territories (stress MBF: 26 ± 12% vs.25 ± 9% vs.24 ± 9%, p = 0.62; MPR: 31 ± 22% vs. 27 ± 23% vs.27 ± 24%, p = 0.79 respectively). There was no significant cyclical variation in rest MBF in any territory (Table 2).

#### Reproducibility

Intra-observer CoVs for stress MBF, rest MBF, MPR and MCV in stress MBF were 9%, 11%, 15% and 10%, respectively. Corresponding CoVs for inter-observer reproducibility were 14%, 16%, 18% and 12% respectively.

## Discussion

The main findings of this study are 1) quantitative perfusion CMR can track physiological changes in myocardial perfusion throughout the cardiac cycle; 2) estimates of stress MBF and MPR in healthy volunteers show significant cyclic variation with successive reductions throughout systole and an increase in diastole; 3) the greatest interval change in stress MBF and MPR occurs between end-systole and early-diastole; 4) there is no significant cyclic variation in rest MBF estimates.

The MBF values derived in the present study are comparable to values from PET studies and the previous CMR literature [16,17]. For example, in a large study of 160 healthy men and women with PET, the mean resting MBF was 0.98 ± 0.23 ml/min/g (range 0.59-2.05 ml/min/g) and the mean stress MBF was 3.77 ± 0.85 ml/min/g (range 1.85-5.99 ml/min/g) [17]. The finding of significant cyclic variation in stress MBF and MPR is consistent with the expected physiology relating to phasic changes in myocardial tension. In vivo animal studies directly visualising the microcirculation with angiography show significant compression of intramyocardial vessels during systole [18]. In humans, coronary flow patterns across the cardiac cycle have been directly measured with intracoronary pressure wires. Davies *et al.* showed that blood flow in the left coronary artery (LCA) is predominantly diastolic i.e. when myocardial tension is low [19]. During systole, they found a dominant forward-travelling pushing wave, which is reflected when reaching the microvascular bed because of the higher myocardial tension, and this results in virtually no forward flow, or even retrograde flow. Therefore, our observation that MBF estimates successively fall throughout systole and increase in diastole is consistent with these described coronary flow patterns.

The greatest interval change in stress MBF and MPR was seen between end-systole and early-diastole (Table 2, Figures 1, 2 and 3). This is in keeping with invasive studies that show LCA inflow velocity peaks in early-diastole [19]. It is also consistent with an observation by Davies *et al.* that there is a transient secondary forward-travelling pushing wave in early diastole seen in LCA waveforms. This secondary wave coincides with closure of the aortic valve and accelerates blood further towards the myocardium.

Heller *et al.* showed that arterial waveforms in the proximal RCA have significantly less phasic variation because right ventricular peak systolic pressure is much lower than aortic peak systolic pressure and because the right ventricular wall offers less mechanical compressive resistance [20]. However, they also showed that distal RCA branches (posterior descending and posterolateral coronary arteries) show the same diastolic dominance as the left coronary system as they subtend LV myocardial segments subjected to the same phasic changes in myocardial tension as the rest of the LV. Consistent with this observation, we found no significant differences in MCV in stress MBF between the left coronary (LAD and LCX) and RCA perfusion territories - and they showed the same pattern of phasic variation across the 5 time points(Table 2).

Three previous quantitative perfusion CMR studies (including 1 utilising 3D data acquisition) have also shown significant differences in stress MBF and MPR according to phase, with significantly higher values in diastole [9-11]. A recent semi-quantitative study also found steeper myocardial time-intensity curves in diastole compared to systole [21]. However, all of these previous studies have been limited to the assessment of myocardial perfusion at only two different time points in the cardiac cycle. The current study is the first to assess variation in MBF estimates *throughout* the cardiac cycle in humans and determine the overall trend between 5 selected time points. Recently, cyclic changes in myocardial perfusion have also been examined in rats (n = 7) by Troalen *et al.* using a novel steady-pulsed arterial spin labelling (ASL) approach to map MBF [22]. Dynamic MBF maps were obtained with an extremely high temporal resolution (6 ms) offering even more comprehensive coverage throughout the cardiac cycle than in our study. However, the acquisition time using this technique in rats was approximately 12 min (at a heart rate of 400 bpm), preventing application of this method to humans where even longer acquisition times would be required. Furthermore, the use of myocardial ASL in humans to quantify MBF remains limited by inadequate signal-to-noise ratio (SNR) efficiency, high physiological noise, and timing restrictions related to cardiac and respiratory motion [23]. Nonetheless, this small study in rats showed a similar phasic variation in stress MBF estimates, which peaked in diastole and steadily fell in systole with an overall mean MCV of 18 ± 8%.

In our study, there was no significant cyclic variation in rest MBF estimates (Table 2, Figure 1). This is in keeping with the result of the 3 previous volunteer and patient studies, which also showed no significant difference between rest MBF estimates in systole and diastole [9-11]. A fourth study assessing semi-quantitative measures of resting myocardial perfusion also found no significant difference between systole and diastole [12]. A possible explanation for a lack of cyclic variation in resting MBF is sufficient autoregulation in the microvascular network at rest, which is only overcome in the stress state by adenosine-induced maximal hyperemia or significant tachycardia. Only the recent study in rats utilising ASL showed a significant cyclic variation in resting MBF, but this was under the influence of isoflurane anaesthesia which is known to induce coronary vasodilatation and therefore not representative of a true physiological resting state [22,24]. Additionally, the resting heart rate in rats is significantly higher than in humans (322 ± 43 bpm in the study by Troalen *et al.*) and therefore these findings are not necessarily translatable to human physiology [22].

Finally, we found a strong negative linear correlation between MCV and peak heart rate during stress (Figure 5). Notably, this observation was also seen in the aforementioned study in rats by Troalen *et al.* [22]. A possible explanation is the capacitive property of the myocardial vascular system due to an abundant capillary network. The latter serves to dampen the rapid fluctuations in pressure seen with increasing heart rates in order to maintain a steady downstream blood flow – the so-called ‘windkessel’ effect [25]. At lower heart rates the effects of phasic myocardial tension are relatively unopposed by capacitance and thus a greater MCV is seen which is in keeping with our findings.

One implication of our data is that phasic variation in CMR estimates of MBF should be considered an important limitation of quantitative 2D perfusion CMR studies – particularly if inter-slice or longitudinal comparisons are made. Similarly, for quantitative 3D perfusion CMR studies (in which the phase of acquisition of all slices can be specifically chosen) the cardiac phase of acquisition should be stated. Moreover, these findings call for the standardisation of acquisition techniques if quantitative perfusion CMR is to be more widely adopted.

In summary, we have demonstrated that significant phasic differences in MBF estimates quantified with CMR are seen not only at polar ends of the cardiac cycle but also *throughout* the cardiac cycle. Considering the nature of coronary hemodynamics, cyclic MBF changes may reveal new physiological information because they are a function of coronary flow, myocardial contraction and microvascular condition. The response of the coronary circulation to phasic pressure changes is modulated by an array of autoregulatory mechanisms and endothelial factors which are dependent on the integrity and function of the microvasculature [26]. It is feasible therefore that increased MCV may be a marker of microvascular disease in conditions such as hypertension, diabetes or pre-clinical coronary artery disease (CAD). Therefore, using CMR to assess MBF throughout the cardiac cycle and determine parameters such as MCV, may be useful in the future assessment of diseases known to alter microvascular function - but further studies in these disease states are clearly needed.

### Study limitations

We acknowledge this was a small study limited to healthy volunteers but was nonetheless important as proof of principle. A larger study in well-defined patient groups is the next logical step in order to assess if the same cyclic pattern is seen in patients with reduced MBF e.g. with CAD; or if MCV is exaggerated by diseases causing endothelial dysfunction due to impaired coronary autoregulation.

The spatio-temporal undersampling method required to accelerate perfusion data acquisition is sensitive to respiratory motion, cardiac arrhythmia and low-pass temporal filtering - all of which pose challenges to quantitative assessment. Low-pass temporal filtering in particular may have led to underestimation of MBF.

To obtain systolic and diastolic perfusion data in the same location and within the same acquisition, this study was limited to the assessment of a single mid-ventricular section. This was a technical necessity and meant we could not assess phasic differences in apical and basal myocardial segments, which may behave differently from the mid-ventricle. Future studies with more advanced acceleration and 3D perfusion data acquisition are needed to address these issues - but these strategies also come with additional challenges for absolute MBF quantitation.

Finally, the model used for estimating MBF assumes a linear relationship between signal and contrast agent concentration i.e. ignoring saturation effects in the LV blood pool, which can lead to underestimation of MBF [27]. Proposed solutions include the use of a non-linear signal model combined with pre-contrast T1-mapping and/or the use of a small pre-bolus to measure the AIF. However, such methods add further complexity to data acquisition and post-processing - and therefore neither was used in this study. Furthermore, there is currently no evidence that either of these potential solutions actually leads to improved diagnostic accuracy in the clinical setting (e.g. for the detection of CAD). In fact the only study directly addressing this question came to the opposite conclusion i.e. the use of a pre-bolus AIF was found to reduce diagnostic accuracy compared to a single-bolus approach [28]. Additionally, our findings are based on intra-individual comparisons and the relative changes in perfusion values throughout the cardiac cycle, and therefore underestimation in absolute MBF due to saturation effects is less relevant.

## Conclusions

Quantitative perfusion CMR can be used to non-invasively track cyclical variations in MBF *throughout* the cardiac cycle. In this study, estimates of MBF followed the expected physiological trend, peaking at end-diastole and falling steadily through to end-systole. This technique may be useful in future physiological or pathological studies of coronary flow and microvascular function.

## Abbreviations

AIF: Arterial input function
CMR: Cardiovascular magnetic resonance
CoV: Coefficient of variation
*k-t*: *k*-space and time
LAD: Left anterior descending
LCX: Left circumflex
MCV: Maximal cyclic variation
MBF: Myocardial blood flow
MPR: Myocardial perfusion reserve
RCA: Right coronary artery
ROI: Region of interest
